# The Direct and Indirect Impact of SARS-CoV-2 Infections on Neonates: A Series of 26 Cases in Bangladesh

**DOI:** 10.1097/INF.0000000000002921

**Published:** 2020-10-06

**Authors:** Senjuti Saha, ASM Nawshad Uddin Ahmed, Probir Kumar Sarkar, Mohammed Rizwanul Ahsan Bipul, Kinkar Ghosh, Sheikh Wasik Rahman, Hafizur Rahman, Yogesh Hooda, Nafiz Ahsan, Roly Malaker, Mohammad Saiful Islam Sajib, Mohammad Shahidul Islam, Ataul Mustufa Anik, Sudipta Saha, Naito Kanon, Maksuda Islam, Davidson H. Hamer, Ruhul Amin, Mohammod Shahidullah, Samir K. Saha

**Affiliations:** From the * Child Health Research Foundation, Dhaka, Bangladesh; † International Health, Johns Hopkins Bloomberg School of Public Health, Baltimore, Maryland; ‡ Dhaka Shishu (Children) Hospital, Dhaka, Bangladesh; § Bangladesh Institute of Child Health, Dhaka, Bangladesh; ¶ MRC-Laboratory Molecular Biology, Cambridge, United Kingdom; ‖ Li Ka Shing Knowledge Institute, St. Michael’s Hospital, Toronto, ON, Canada; ** Department of Global Health, Boston University School of Public Health, Boston, Massachusetts; †† Section of Infectious Disease, Department of Medicine, Boston University School of Medicine, Boston, Massachusetts; ‡‡ National Emerging Infectious Disease Laboratory, Boston University, Boston, Massachusetts; §§ Department of Neonatology, Bangabandhu Sheikh Mujib Medical University, Dhaka, Bangladesh.

**Keywords:** SARS-CoV-2, COVID-19, neonates, low- and middle-income country, Bangladesh

## Abstract

**Background:**

The impact of SARS-CoV-2 on neonates remains largely unknown in low- and middle-income countries (LMICs). We provide an epidemiologic and clinical report of SARS-CoV-2 infections in neonates hospitalized in Bangladesh.

**Methods:**

Outborn neonates admitted to Dhaka Shishu Hospital, a tertiary-care referral hospital, between 29 March and 1 July were screened for SARS-CoV-2. We reviewed clinical data, including chest radiograph and laboratory reports, and conducted SARS-CoV-2 genome sequencing. Patients were followed-up for 27–75 days. A subset of caregivers was also tested.

**Results:**

Of 83 neonates tested, 26 were positive (median age 8 days). Most neonates were admitted with diagnosis unrelated to SARS-CoV-2: 11 presented with serious non-communicable diseases, 7 with early-onset sepsis, 5 with late-onset sepsis and 2 with pneumonia. In 3 of 5 chest radiograph, infiltrates and ground-glass or patchy opacities were noted. Two neonates developed metabolic acidosis, one developed disseminated intravascular coagulation. Most SARS-CoV-2 positive neonates were referred to government-designated COVID-19 hospitals, leading to gaps in treatment. Twenty-three neonates could be followed-up: 12 were healthy, 8 died and 3 were still seeking medical care. Of 9 caregivers tested, 8 were positive.

**Conclusions:**

SARS-CoV-2 may have serious adverse effects on children born in LMICs. The virus likely contributed directly to two deaths, but the remaining 6 neonates who died had serious comorbidities. Positive SARS-CoV-2 test results led to gaps in immediate clinical care for other morbidities, which likely contributed to adverse outcomes. This case series emphasizes the need to understand COVID-19 in neonates in LMICs and its indirect impacts.

## Abstract 2

Supplemental Digital Content is available in the text.

The ongoing SARS-CoV-2 pandemic has led to over 25 million cases and caused at least 854,000 deaths worldwide as of August 31, 2020, 15% of which have been reported from South Asia.^1^ However, limited data are available on the epidemiology of the disease in this region. In addition, numerous reports describing COVID-19 in children are becoming available, but little is known about the clinical presentation and outcomes in children in South Asia. The evidence gap is even wider in case of neonates (<29 days old).

Although some studies suggest that children may be less vulnerable to SARS-CoV-2 infections than adults, with the exception of pediatric multisystem inflammatory syndrome,^2^ this evidence is equivocal and evolving as non-pharmaceutical interventions change (eg, school closures).^3^ There is a lack of evidence relating to neonates specifically. The first case of neonatal SARS-CoV-2 infections was identified on February 2, 2020 in China.^4^ Since then, approximately 70 cases have been described, and symptoms ranged from mild to severe, and no deaths have been reported thus far.^5–9^ However, little is known about infection amongst neonates in South Asia, which already shares a disproportionately high burden of global neonatal morbidity and mortality.^10,11^ By August 31, 2020, there had been over 43 million infections and 77,000 deaths in South Asia, but only 2 sporadic reports on the impact of SARS-CoV-2 on neonates in this region had been published.^6,12^

In this study, we report a series of 26 cases of SARS-CoV-2 infections in neonates who presented at Dhaka Shishu Hospital (DSH), the largest pediatric tertiary care, referral hospital in Bangladesh. We outline the hospital clinical records, chest radiograph, hematologic, biochemical and molecular tests, genome sequences and follow-up data for these cases. Our findings highlight the direct and indirect impact of SARS-CoV-2 infections on neonates in South Asia and provide a snapshot of the presentation of these cases.

## METHODS

### Study Site, Design and Participants

This observational prospective study was conducted in DSH, which provides primary to tertiary care to children and treats 37% of patients free of cost. This is the largest referral hospital for children in Bangladesh, and very sick children requiring immediate or intense care are referred to this hospital.

Starting in March 2020, the government of Bangladesh established designated COVID-19 hospitals across the country, but for the duration of this study, DSH was not one of them. However, DSH continued to admit children requiring medical attention. Children with COVID-19 symptoms were admitted in an isolation ward till SARS-CoV-2 tests were conducted, and results were attained. In addition, children admitted at DSH, who required surgical procedures or ICU care, were also tested for SARS-CoV-2 for screening. Cases that tested positive were referred to a COVID-19 hospital.

This study recruited neonates (0–28 days) who presented at DSH between 29 March and 1 July and tested positive for SARS-CoV-2. Between 9 June and 1 July, nasopharyngeal specimens were also collected from the immediate caregiver of these neonates.

### Specimen Collection and Testing

Nasopharyngeal specimens were collected by trained physicians or nurses using a COPAN FLOQswab (503CS01; COPAN Diagnostics, Brescia, Italy), which was immediately transferred into a 15 mL tube containing 1 mL of 1X RNA shield (D7005; Zymo, Irvine, CA).

All tests were performed at the Child Health Research Foundation that runs 4 WHO sentinel laboratories, including the laboratory in DSH,^13^ and is a government-designated testing center for COVID-19. RNA from all samples was extracted within 24–48 hours of collection and tested for the presence of SARS-CoV-2 using the TaqPath COVID-19 RT-PCR Kit (A7817; Thermo Fisher Scientific, Waltham, MA).

### Genome Sequencing

Viral RNA was extracted from nasopharyngeal specimens using the Quick-RNA/DNA Microprep Extraction Kit (D7005; Zymo), and was converted to cDNA using ProtoScript II (E6560; New England Biolabs, Ipswich, MA). Libraries prepared following the ARTIC pipeline,^14^ were sequenced on an Illumina iSeq100 sequencer using 150-nucleotide paired-end sequencing. SARS-CoV-2 reads were recovered by mapping the raw reads against the reference sequence (GenBank accession no. MN908947.3, https://github.com/czbiohub/sc2-msspe-bioinfo).^15,16^ Consensus sequences of the 9 SARS-CoV-2 genomes were uploaded on GISAID^17^ and subsequently analyzed using NextStrain (https://nextstrain.org/).^18^

### Clinical Data Collection and Analysis

Demographic and clinical data were prospectively collected with a standardized form for 25 of 26 cases. The hospital file of the first identified SARS-CoV-2 neonatal case was misplaced, and therefore only partial data could be retrieved retrospectively through telephone interviews. Laboratory data for all cases were collected from the COVID-19 database of the laboratory. Outcomes of the positive cases were followed-up over telephone through July 28, 2020, upon consent, using structured questionnaires.

### Ethical Considerations

All protocols were approved by the National Research Ethics Committee, Bangladesh Medical Research Council, and the ethical review board of the Bangladesh Institute of Child Health. Samples from suspected COVID-19 patients were collected for clinical care and diagnostic testing at the discretion of the attending healthcare providers.

## RESULTS

**FIGURE 1.**
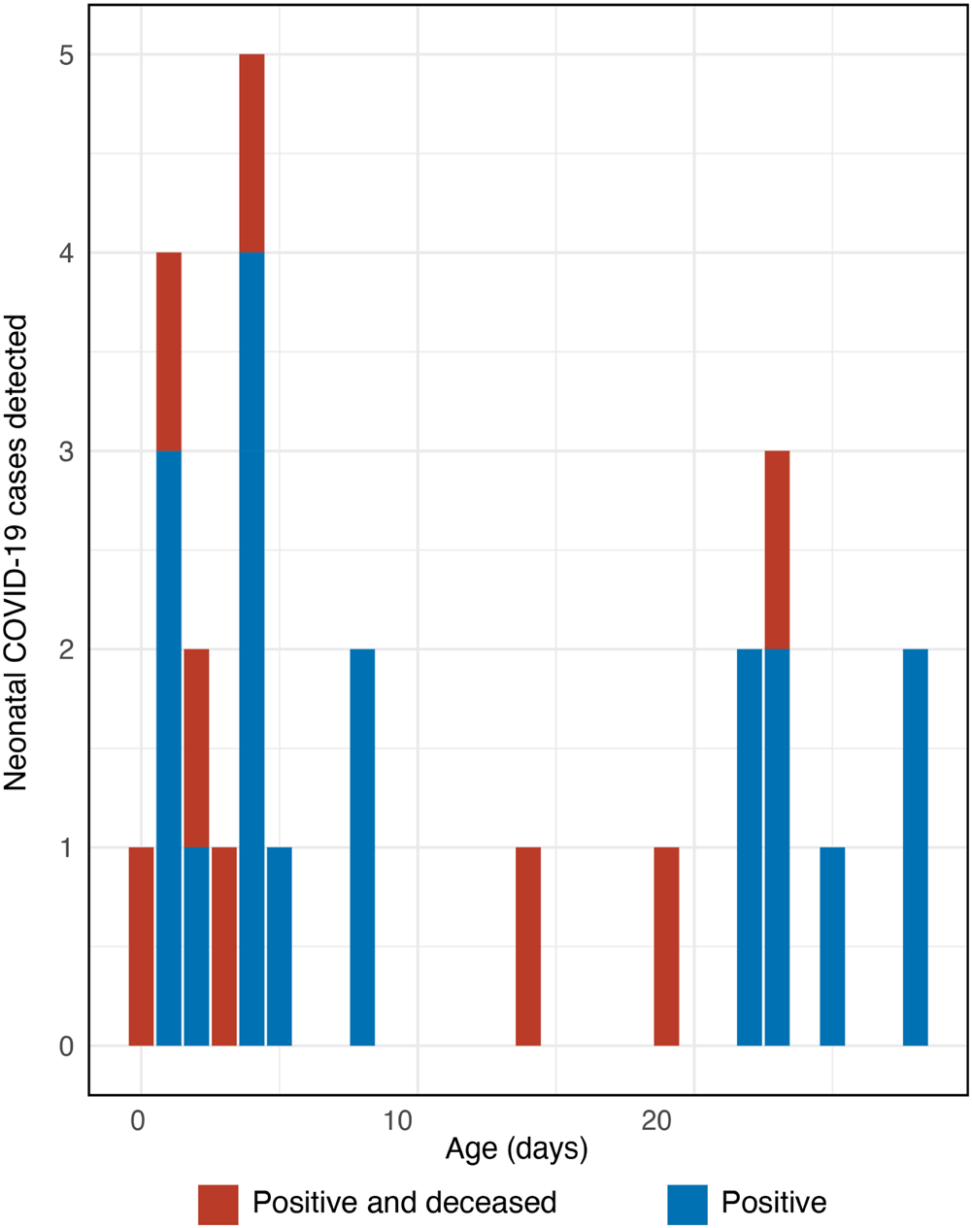
Age distribution and outcome of neonatal cases of SARS-CoV-2.

Between March 29, 2020 and July 01, 2020, nasopharyngeal swab specimens were tested for SARS-CoV-2 from 83 neonates with various medical presentations. Twenty-six neonates (31%) tested positive and 16 of 26 (62%) were boys. The median age of the positive cases was 8 days and 14 babies (53%) were in their first 5 days of life (Figure 1). No other etiologies were identified in these 26 cases. All babies were born at term, and mean weight at admission was 2.9 kg. Cases were followed-up through July 28, 2020. Four babies died at the hospital, 18 were referred to hospitals designated to treat COVID-19 by the Government of Bangladesh. Of the 22 children who left DSH alive, 19 were followed-up; 12 were healthy, 3 were still seeking medical care and 4 had died.

**FIGURE 2.**
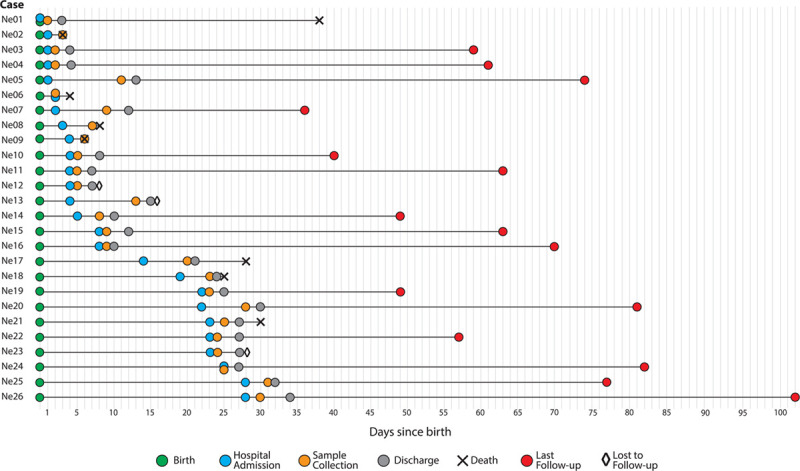
Overview of the key events of the 26 SARS-CoV-2 neonatal infections. This includes dates of hospital admission and other key events for each case during the course of this surveillance study.

The main clinical and biologic characteristics, treatments and outcomes of the 26 patients at hospital admission are summarized in Table 1. Key events are illustrated in Figure 2. Detailed history of each case is provided in the Supplemental Digital Content 1, http://links.lww.com/INF/E137. Of the 25 cases for which detailed hospital data were available, 7 cases presented with symptoms of early-onset neonatal sepsis (EONS), 5 with late-onset neonatal sepsis (LONS), 2 with pneumonia and 11 with serious non-communicable diseases.

**TABLE 1.** Clinical and Epidemiologic Characteristics, Treatment and Outcome of 26 Neonatal Cases of SARS-CoV-2 Infections

ID	Age (Days)/Sex	Diagnosis	Treatment	Hospital Duration (Days)	Hospital Outcome (Age at Death)	Follow-up Outcome (Age at Follow-up/Death)
Ne01	0/f	Ruptured myelomeningocele	Ceftriaxone	3	Referred	Died (38)
Ne02	1/f	PNA with HIE stage 2 and congenital pneumonia	Ceftazidime, amikacin, phenobarbitone, Sup 0_2_	2	Died (3)	—
Ne03	1/f	Ruptured myelomeningocele	Surgical repair, ceftazidime, gentamycin, paracetamol	3	Referred	Healthy (59)
Ne04	1/m	Anocutaneous fistula	Anoplasty, ceftazidime, metronidazole	3	Referred	Healthy (61)
Ne05	1/f	Ruptured occipital encephalocele	Excision and repair of occipital region, ceftriaxone, flucloxacillin, gentamicin, phenobarbitone	12	Referred	Healthy (74)
Ne06	2/m	EONS with anorectal malformation	Nasogastric suction, ceftazidime, amikacin, metronidazol	1	Died (4)	—
Ne07	2/m	PNA with HIE stage 2, pneumonia and EONS	Ceftazidime, amikacin, meropenem, netilmicin, phenobarbitone, Sup 0_2_	10	LAMA	Sick (36)
Ne08	3/f	EONS with disseminated intravascular coagulation	Ceftazidime, amikacin, meropenem, vancomycin, dopamine, dobutamine, Sup 0_2_	5	Died (8)	—
Ne09	4/m	EONS, pneumonia, and severe metabolic acidosis	Ceftazidime, amikacin, dopamine, sodium bicarbonate, Sup 0_2_	2	Died (6)	—
Ne10	4/f	Ruptured myelomeningocele	Ceftriaxone, gentamycin	4	Referred	Sick (40)
Ne11	4/f	EONS	Ceftazidime, amikacin, Sup 0_2_	3	Referred	Healthy (63)
Ne12	4/m	EONS and neonatal jaundice	Ceftazidime, phototherapy	3	Referred	LTFU
Ne13	4/m	PNA with HIE stage 2, EONS and pneumonia	Ceftazidime, amikacin, meropenem, netilmicin, piperacillin + tazobactam, furosemide, dobutamine, phenobarbitone, Sup 0_2_	11	Referred	LTFU
Ne14	5/f	EONS and neonatal jaundice	Ceftazidime, amikacin, phenobarbitone, Sup 0_2_	5	Referred	Healthy (49)
Ne15	8/m	Acute kidney injury due to obstructive uropathy	Ceftazidime, meropenem, sodium bicarbonate, calcium gluconate	4	Referred	Sick (63)
Ne16	8/m	LONS	Ceftazidime, amikacin	2	Referred	Healthy (70)
Ne17	14/m	Posterior urethral valve with bilateral hydroureteronephrosis (grade-3) with bilateral TEV	Ceftazidime, amikacin, meropenem, metronidazole, frusemide, calcium gluconate, sodium bicarbonate	7	Referred	Died (28)
Ne18	19/m	Congenital heart disease, LONS and metabolic acidosis	Meropenem, amikacin, aminophylline, phenobarbitone, Sup 0_2_	5	Referred	Died (25)
Ne19	22/m	LONS	Ceftazidime, amikacin, Sup 0_2_	3	Referred	Healthy (49)
Ne20	22/m	LONS	Ceftriaxone, amikacin, phenobarbitone	8	Discharged	Healthy (81)
Ne21	23/m	Congenital heart disease and LONS	Ceftazidime, meropenem, dobutamine, furosemide, calcium gluconate, Sup 0_2_	4	Referred	Died (30)
Ne22	23/m	Epistaxis due to local trauma	Mupirocin 2% topical ointment	4	Referred	Healthy (57)
Ne23	23/m	Pneumonia	Ceftazidime, amikacin, Sup 0_2_	4	Referred	LTFU
Ne24	25/f	LONS and pneumonia	Ceftriaxone, amikacin, Sup 0_2_	2	Referred	Healthy (82)
Ne25	28/f	LONS and pneumonia	Meropenem, amikacin	4	LAMA	Healthy (77)
Ne26	28/m	NA	NA	6	Discharged	Healthy (102)

All ages are in days.

TEV indicates talipes equinovarus; PNA, perinatal asphyxia; AGA, appropriate gastrointestinal age; RDS, respiratory distress syndrome; LTFU, lost to follow-up; LAMA, left against medical advice; NA, not available.

### Neonates Presenting With Early Onset Neonatal Sepsis

**FIGURE 3.**
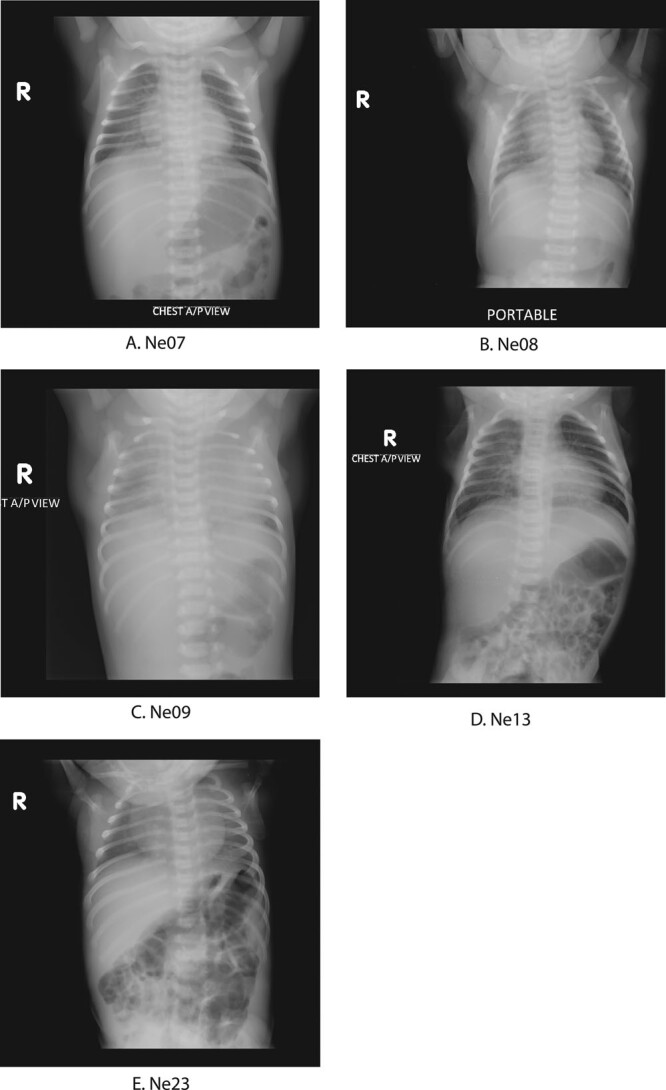
Chest radiograph images of 5 neonates with SARS-CoV-2 infections. A: Chest radiograph of Ne07 showed patchy opacities in right lower lobe suggesting pneumonia. B: Chest radiograph of Ne08 looked normal. C: Chest radiograph of Ne09 showed bilateral ground-glass opacity indicative of SARS-CoV-2. D: Chest radiograph of Ne13 showed few patchy opacities in the right lower perihilar region indicating nonspecific inflammatory lesion in right lower zone. E: Chest radiograph of Ne23 looked normal.

Seven babies, Ne07, Ne08, Ne09, Ne11, Ne12, Ne13 and Ne14, presented at the hospital with clinical signs of EONS. Of specific note is patient Ne09, who also showed signs of pneumonia, and his chest radiograph showed bilateral ground-glass opacity indicative of SARS-CoV-2. The baby required supplemental oxygen, developed severe metabolic acidosis (see Table, Supplemental Digital Content 2, http://links.lww.com/INF/E138) and died at the age of 6 days. Similarly, patient Ne08 had respiratory distress, required supplemental oxygen and showed signs of disseminated intravascular coagulation and eventually died on the sixth day of hospitalization, at the age of 8 days. Patients Ne12 and N14, who were admitted with neonatal jaundice, and Ne11 were referred to COVID-19 hospitals. Ne12 was lost to follow-up and Ne14 and Ne11 were doing well during follow-up. Ne07 and Ne13 were admitted with perinatal asphyxia and hypoxic-ischemic encephalopathy (HIE) stage 2 along with EONS. Chest radiograph of Ne07 showed patchy opacities in the right lower lobe suggesting pneumonia (Fig. 3A) and chest radio graph of Ne13 showed patchy opacities in the right lower perihilar region indicating nonspecific inflammatory lesion (Fig. 3D). Ne07 was still seeking medical attention during follow-up, and Ne13 was lost to follow-up.

### Neonates Presenting With Late-onset Neonatal Sepsis

Five children were admitted with signs of LONS: Ne16, Ne19, Ne20, Ne24 and Ne25. Among them, 3 patients, Ne16, Ne19 and Ne20 presented with fever; in addition, Ne19 had respiratory distress and Ne20 had convulsions. Ne16 and Ne19 were referred to a COVID-19 hospital after 2 and 3 days, respectively, and Ne20 was discharged after 8 days. All 3 patients were doing well during follow-up. Patients Ne24 and Ne25 showed signs of pneumonia with bilateral crepitations during admission. Ne24 was referred to a COVID-19 hospital after 2 days, and family of Ne25 left after 4 days. During follow-up, both these children were also doing well.

### Neonates Presenting With Pneumonia

Patient Ne02 and Ne23 presented with signs of pneumonia. Ne02 was admitted with perinatal asphyxia and HIE stage 2, and congenital pneumonia with bilateral crepitations. The child died after 2 days, at the age of 3 days. Patient Ne23 presented with fever, respiratory distress and bilateral crepitations and also required supplemental oxygen. After 4 days at DSH, the baby was referred to a COVID-19 hospital and was lost to follow-up.

### Neonates Presenting With Non-communicable Diseases

Eleven cases presented with serious non-communicable diseases at admission. These cases were screened for SARS-CoV-2 before surgery, before admission into the critical-care unit or due to contact history. Ne01, Ne03 and Ne10 were admitted with ruptured myelomeningocele, Ne04 had anocutaneous fistula, Ne05 with ruptured occipital encephalocele, Ne06 with anorectal malformation, Ne15 and Ne17 with obstructive uropathy, Ne18 and Ne21 with congenital heart disease, and Ne22 with epistaxis due to fall from height.

For 3 cases (Ne03, Ne04, Ne05) emergency surgeries were performed soon after hospital admission before the SARS-CoV-2 test results were obtained. For postoperative care, they were referred to COVID-19 hospitals. All 3 children were reported to be doing well during follow-up. Three other cases that also required surgeries (Ne01, Ne10, Ne15) were referred to COVID-19 hospitals immediately after SARS-CoV-2 test results were obtained. Ne01 died at another hospital at the age of 38 days; Ne10 had 1 surgery and was reported to be waiting for a second surgery during a telephone follow-up 40 days of testing positive; Ne15 was also still sick and seeking medical care.

Ne06, admitted with anorectal malformation, died the day after admission due to sepsis. Ne18 and Ne21, both of whom were admitted due to congenital heart disease, developed signs of respiratory distress. Ne18 also developed LONS and metabolic acidosis (arterial blood gas results are shown on Table, Supplemental Digital Content 2, http://links.lww.com/INF/E138) and was referred to a COVID-19 designated hospital; however, the family did not seek care at any other hospital and the child died at home at the age of 25 days. Ne21 was also referred to a COVID-19 hospital after 4 days, and died at the age of 30 days.

Ne17 was admitted with posterior urethral valve with bilateral hydroureteronephrosis, with bilateral talipes equinovarus. After 8 days, the patient was referred to a COVID-19 hospital, where he died at the age of 28 days. Ne22 was admitted with epistaxis due to fall, discharged after 4 days, and was doing well during follow-up. Overall, cases referred to designated COVID-19 hospitals due to a SARS-CoV-2 positive result, despite other serious morbidities that required immediate and constant care, suffered from delays and gaps in treatment. Some families went home instead of seeking care at another hospital that also led to adverse outcomes.

### Maternal SARS-CoV-2 Infection

Twenty of the 26 families could be interviewed to determine potential exposure to SARS-CoV-2, and in total, 6 mothers reported experiencing COVID-19 related symptoms in the days before delivery. Immediate caregivers from 9 neonates (8 mothers and 1 grandmother) provided nasopharyngeal swab specimens during the time of specimen collection from the neonates; 8 of them tested positive for SARS-CoV-2 (see Table, Supplemental Digital Content 2, http://links.lww.com/INF/E138), and 4 of them exhibited COVID-19 symptoms. Amongst the mothers who were not tested, 2 reported symptoms related to COVID-19.

### Genome Sequencing and Geographic Distribution

Genome sequences were obtained from 9 neonatal cases that had cycle threshold value of less than 25 for *nucleoprotein* gene (specimens with high viral RNA concentration). These clustered with other Bangladeshi isolates in clades 20A, 20B and 20C (see Figure, Supplemental Digital Content 3, http://links.lww.com/INF/E139), and no specific distinct cluster was seen for these cases. The residential locations of the cases were distributed across the country (see Figure, Supplemental Digital Content 4, http://links.lww.com/INF/E140).

## DISCUSSION

There are emerging reports of SARS-CoV-2 infection in children and some studies have also indicated that COVID-19 in children may present with atypical symptoms.^2,19,20^ This is of specific concern to low- and middle-income countries (LMICs), where newborns are already the most vulnerable population, and several reports indicate the decline in essential care due to the pandemic.^21^

This study, to our knowledge, presents the largest case series of SARS-CoV-2 infections in neonates to date. We screened 83 babies admitted at a tertiary care pediatric hospital, and 26 (31%) tested positive for SARS-CoV-2 by RT-qPCR. We recorded a wide spectrum of presenting signs and symptoms. These included fever, respiratory distress, gastrointestinal symptoms and a range of non-communicable morbidities like perinatal asphyxia and HIE that are commonly reported as neonatal causes of hospitalization and deaths in this region. Three of the 5 chest radiographs available showed changes consistent with pneumonia. Previous publications on neonatal COVID-19 cases have identified fever, respiratory distress and ground-glass opacity in chest radiographs as the most common symptoms in neonates;^7,22^ presentation of gastrointestinal symptoms have also been noted.^5^

Of the 8 babies who died, 6 had serious co-morbidities—ruptured myelomeningocele, perinatal asphyxia and HIE stage 2 and congenital pneumonia, anorectal malformation, posterior urethral valve with bilateral hydroureteronephrosis, and congenital heart disease. These morbidities have been associated with high mortality in LMICs,^11^ and therefore it is difficult to determine the role of SARS-CoV-2 in death. However, it is clear that in both children and adults, severity of and mortality due to COVID-19 are amplified in patients with specific preexisting morbidities.^23,24^ This might also be the case in neonates, where SARS-CoV-2 infection might aggravate the health of already vulnerable children.

In 2 patients who died, Ne08 and Ne09, SARS-CoV-2 infection was likely the key contributor. Ne09 was admitted with EONS and developed severe metabolic acidosis; chest radiograph showed bilateral ground-glass opacity. Both the bilateral ground-glass opacity and metabolic acidosis have been reported in severe COVID-19 patients.^25–27^ Ne08 presented with respiratory distress and was diagnosed with EONS with disseminated intravascular coagulation, which has been reported in severe COVID-19 patients.^28^ Both the babies died before they reached the ninth day of life. These 2 cases provide evidence that SARS-CoV-2 infections may lead to systemic disease with adverse outcomes in neonates.

In addition to the direct impact of SARS-CoV-2 on neonates, this snapshot of vulnerable neonates born in LMICs in the midst of the COVID-19 pandemic and the loss of care or attention, depict the indirect impacts this virus can have on neonatal health. For example, the family of baby Ne10 is still waiting for a surgery, and baby Ne01 died at the age of only 38 days when the family was still waiting for a surgery. Such surgeries are routinely performed at DSH but due to the pandemic (and sometimes due to infections of health care providers themselves), cases had to be referred to general COVID-19 designated hospitals. Such delays in medical treatment are likely to have serious outcomes in the near future.

To confirm the positive results seen in the RT-qPCR assays, SARS-CoV-2 genome sequences were obtained from 9 cases with high viral loads. Considering that the SARS-CoV-2 genomes belonged to different clades, and tests were conducted within 72 hours of admission in 19 (73%) cases, the virus was likely acquired before presentation at DSH, either in the household or at the hospital of birth. To further investigate potential sources of infections, we tested samples from 9 caregivers, 8 of whom tested positive. Of all 20 families interviewed, 6 mothers reported experiencing symptoms related to COVID-19 in the days before child delivery. While it was out of the scope of this study to further examine vertical or horizontal transmission from mother, this does suggest that neonatal infections are likely to increase if SARS-CoV-2 continues to spread in the population, and preventative policies need to be designed and instituted immediately to protect children from acquiring the infection in this critical period of their development.

The findings of this study should be considered within the context of several limitations. First, DSH does not have a maternity facility and hence neonates were only admitted here if they had serious conditions, biasing our findings towards the most severe outcomes. Our surveillance did not include asymptomatic SARS-CoV-2 positive neonates and their outcomes. Second, as also alluded to above, most of the babies that tested positive were referred to government-designated COVID-19 hospitals and this transfer led to a gap in their treatment that could have negatively impacted their outcome (4 babies died after leaving DSH). Third, due to reduced hospital capacity because of COVID-19, chest radiographs were only performed on 5 cases, and limited laboratory tests were conducted, inhibiting more nuanced understanding of the inflammatory state of the neonates. Finally, the study only followed up patients for 27–75 days after testing positive. It has been reported that children with exposure to SARS-CoV-2 can have serious long-term effects like multisystem inflammatory response months after the infection.^2^

Despite these limitations, this study has important lessons for the design of future studies looking at SARS-CoV-2 infections in children, specifically neonates. First, except for 2 cases that had typical COVID-19 symptoms, neonates presented with one or more comorbidities often seen in South Asia and other LMICs. Future studies should take this into consideration when designing their inclusion criteria to capture SARS-CoV-2 infections in this age group. The effects of COVID-19 may manifest or become apparent after other presenting comorbidities have been addressed. Second, half of SARS-CoV-2 infections were identified within the first 5 days of life indicating that patients should be screened very early to capture SARS-CoV-2 infections. Finally, while it is difficult to assign SARS-CoV-2 as the cause of death due to multiple comorbidities present in these cases, including several serious noninfectious clinical features, our study provides evidence that neonates with SARS-CoV-2 infections can be adversely affected, whether directly by the virus, or indirectly due to lack of optimal care as a result of a positive test. We recommend that policymakers take this into account and provide specific attention to neonates with SARS-CoV-2 infections when designing treatment policies for COVID-19 treatment.

## ACKNOWLEDGMENTS

We thank the Directorate General Health Services, Government of Bangladesh, and the Institute of Epidemiology, Disease Control, and Research for giving us the opportunity to work as a COVID-19 response team. We thank all personnel at the Child Health Research Foundation involved in the COVID-19 diagnostic, clinical and follow-up teams, including Shumaiya Ferdaus, Shamsun Nahar, Humaira Shusmita, Rumana Khan, Tasmim Lipi, Shamima Tarafder, Asiya Akter, Fatema Akter Kona, Asifa Taslin Shipa, Shelly Akter, Christina Bain, Nasrin Sultana, Apurba Rajib Malaker, Sharmistha Goswami, Nikkon Sarker, Nahidul Islam, Md. Rasel Mahmud, Dipu Chandra Das, Rathindranath Kabiraj, Nusrat Alam, Shuborno Islam, Akibul Hassan Sazal, Zannatul Mauya, Jibon Hossain, Md. Reaz Hossain Khan, Ranjit Sarker, Anik Sarker, Sharif Hasan, Saif Islam, Rahul Mahmud, Jahid Hasan Sheetal, Md. Mizanur Rahman Masum, Solayman Sarker, Ashish Kumar Das, Md. Shohag Alom Bhuiya, Saikat Hossain Khan, Shimul Gain, Purno Biswas, Shanto Bormon, Md. Obaidullah Gain, Ishrat Zahan, Sharmin Jahan, Kanis Fatema, Shahina Tarafder, Khairun Nahar, Razia Sultana, Robi Das, Asifa Jahan Bithi, Sathi Akter, Md. Mahmudul Hasan, Md. Syed Fuad, Md. Imarot Hossain, Taniya Nasrin, Khursheda Afrin Khushi, Zarin Sultana, Sumi Parvin, Monalisa, Fauiya Khanam Luna, Masrufa Akhter, Lima Akter, Uttam Kumar Barui, Shamima Sultana, Md. Yeasin Ali and Md. Zahid Hossain Swapon. We thank our extremely efficient administration and data management team of Rubana Sultana Aflatun, Mahabub Alam, Rahat Fardush, Md Sharif Islam, Said Al Haque and Ruma Dutta. We are especially grateful to Arif M. Tanmoy, Zabed Ahmed, Md. Hasannuzaman for their intellectual guidance and Syed Muktadir al Sium and Afroza Akter Tanni for their technical assistance with sequencing. We thank the Chan Zuckerberg Biohub and the Chan Zuckerberg Initiative, specifically Joseph L. DeRisi, Cristina M. Tato, Vida Ahong, Manu Vanaerschot, Jack Kamm, Joshua Batson, Samantha Hao, Amy Kistler and Katrina Kalantar, for their intellectual support and guidance during genome sequencing and consensus genome building.

## Supplementary Material


