# Supplementary material for: The Direct and Indirect Impact of SARS-CoV-2 Infections on Neonates: A Series of 26 Cases in Bangladesh
Source: Pediatr Infect Dis J. 2020 Oct 6;39(12):e398–405. doi: 10.1097/INF.0000000000002921 (PMC7654949; doi:10.1097/INF.0000000000002921)
Supplement: Supplementary file 1 [file inf-39-e398-s001.docx]

# **Supplemental Digital Content 1.** Case history of 26 neonates with SARS-CoV-2 infections

**Case Ne01:**

A newborn girl, born at term by C-section, was admitted on the day of birth with swelling over the lumbo-sacral region, and with cerebrospinal fluid (CSF) leak. On general examination, the child was afebrile, her pulse was 120 beats/min and she weighed 2 kg. Her respiratory rate was 48 breaths/min (vesicular breath sound, no added sound). The child was diagnosed as a case of ruptured myelomeningocele and was treated conservatively with ceftriaxone and local dressing. A nasopharyngeal swab specimen was collected on the second day of hospital admission to exclude SARS-CoV-2 as a prerequisite for surgery. As the child tested positive for SARS-CoV-2, she was referred to a COVID-19 designated hospital after 3 days. During telephone follow-up the family reported that, the child was admitted to another hospital at the age of 38 days with the diagnosis of pneumonia and died on the same day.

Potential exposures: The child was accompanied to the hospital by her grandmother. Nasopharyngeal swab specimen collected from the grandmother at the same time as that from the child tested positive for SARS-CoV-2. The mother was also symptomatic for COVID-19 but was not tested.

**Case Ne02:**

A 1-day old girl, born at term by vaginal delivery, was admitted with history of delayed cry after birth, and multiple episodes of convulsion. During admission, the child was ill-looking, conscious, afebrile, had poor reflexes and active convulsions. Capillary refill time was <3 s, pulse was 160 beats/min, and she weighed 3 kg. Respiratory rate was 70 breaths/min (vesicular breath sound, bilateral crepitations). Provisional diagnosis was perinatal asphyxia with hypoxic-ischemic encephalopathy stage 2 with congenital pneumonia. She was treated with ceftazidime, amikacin, phenobarbitone and supplemental oxygen.

A nasopharyngeal swab specimen collected on the third day of hospital admission tested positive for SARS-CoV-2. The child died the same day due to perinatal asphyxia with hypoxic-ischemic encephalopathy stage 2 and congenital pneumonia.

Potential exposure: Nasopharyngeal swab specimen collected from the mother at the same time as that from the child tested positive for SARS-CoV-2. The mother was asymptomatic.

**Case Ne03:**

A 1-day old girl born at term by C-section, was brought to DSH with ruptured lumbo-sacral swelling. During antenatal check-up ultrasonogram, the child was diagnosed with bilateral ventriculomegaly. On examination during admission, the child was febrile (99.8^o^F), pulse was 130 beats/min and weighed 3 kg. Her respiratory rate was 42 breaths/min (vesicular breath sound, no added sound). CSF leakage from the lumbosacral region was noted but no abnormality in lower limb movement was observed. Her provisional diagnosis was ruptured myelomeningocele and she was treated with ceftazidime, gentamycin and supp. paracetamol. The myelomeningocele was surgically repaired the day after admission under general anaesthesia.

A nasopharyngeal swab specimen collected on the second day of hospital admission tested positive for SARS-CoV-2. After three days, the child was referred to a COVID-19 designated hospital. During a telephone follow-up at the age of 59 days, the family reported that they did not seek care in any other hospital, and the child currently did not appear to have any health problems.

Potential exposure: Source of exposure to SARS-CoV-2 is unknown. Family members or the immediate caregiver were not tested.

**Case Ne04:**

A 1-day old boy, born at term by vaginal delivery at home, was admitted with a complaint of passage of meconium through a small opening near anus since birth. On examination during admission, he was afebrile, capillary refill time was <3 s, pulse was 124 beats/min, and weighed 2.4 kg. His provisional diagnosis was anocutaneous fistula; anoplasty was performed under general anaesthesia, and the child was treated with ceftazidime and metronidazole.

A nasopharyngeal swab specimen collected on the second day of hospital admission tested positive for SARS-CoV-2. The child was referred to a COVID-19 designated hospital after three days, and during a telephone follow-up at the age of 61 days, the family reported that the child was at home and healthy.

Potential exposure: Source of exposure to SARS-CoV-2 is unknown. Family members or immediate caregiver were not tested.

**Case Ne05:**

A 1-day old girl, born at term by C-section, was admitted with ruptured occipital encephalocele. On examination during admission, the baby was afebrile, alert and active; pulse was 128 beats/min and weighed 3 kg. Her respiratory rate was 36 breaths/min (vesicular breath sound, no added sound).

The child underwent surgery 11 days after admission and was referred to a COVID-19 designated hospital the next day. A nasopharyngeal swab specimen collected the day before the surgery (10 days after admission) tested positive for SARS-CoV-2. During the hospital stay, she was treated with ceftriaxone, gentamicin, flucloxacillin and phenobarbitone. During a telephone follow-up at the age of 74 days, the family reported that the child was currently at home and did not appear to have any health problems.

Potential exposure: Source of exposure to SARS-CoV-2 is unknown. Family members or immediate caregiver were not tested.

**Case Ne06:**

A 2-day old boy was admitted with abdominal distension and no passage of meconium since birth. On examination, there was absence of anal opening, he was afebrile, ill-looking, pulse was 144 beats/min and weighed 3 kg. Respiratory rate was 25 breaths/min (vesicular breath sound, no added sound). The child was diagnosed with anorectal malformation and was treated with ceftazidime, amikacin and metronidazole in addition to nothing per oral and nasogastric suction.

A nasopharyngeal swab specimen collected on the day of hospital admission tested positive for SARS-CoV-2. He died on the second day of admission due to early onset neonatal sepsis with anorectal malformation.

Potential exposure: Source of exposure to SARS-CoV-2 is unknown. Family members or immediate caregiver were not tested.

**Case Ne07:**

A 2-day old boy, born at term by C-section, was admitted with complaints of delayed cry after birth and convulsion since birth. During admission, the child was afebrile, lethargic, pink capillary refill time was <3 s, pulse was 140 beats/min, and weighed 2.5 kg. Respiratory rate was 44 breaths/min (vesicular breath sound, no added sound). His provisional diagnosis perinatal asphyxia with hypoxic-ischemic encephalopathy stage 2. The child developed symptoms of sepsis after admission. He was treated with ceftazidime, amikacin, meropenem, netilmicin and phenobarbitone and supplemental oxygen. Chest X-ray showed patchy opacities in right lower lobe suggesting pneumonia (Fig S5A).

A nasopharyngeal swab specimen collected on the seventh day of hospital admission tested positive for SARS-CoV-2. The child left the hospital after 10 days with final diagnosis of perinatal asphyxia with hypoxic-ischemic encephalopathy stage 2, pneumonia and early onset neonatal sepsis, and during telephone follow-up at the age of 36 days, the child was reported to be still sick by the parents and was seeking medical attention.

Potential exposure: The mother reported history of cough, cold and chest pain during her last month of pregnancy. Nasopharyngeal swab specimen collected at the same time as the child tested negative for SARS-CoV-2 by RT-qPCR.

**Case Ne08:**

A 3-day old girl, born at term by C-section, was admitted with history of delayed crying after birth and respiratory distress. On examination, the child was afebrile, pink, pulse was 130 beats/min, and weighed 2.6 kg. Her respiratory rate was 36 breaths/min (vesicular breath sound, no added sound). The provisional diagnosis was perinatal asphyxia with hypoxic-ischemic encephalopathy stage I. After admission, the baby developed fever (102^o^F), features of sepsis and bleeding from the umbilicus. She was treated with ceftazidime, amikacin, meropenem, vancomycin, dopamine, dobutamine and supplemental oxygen. Chest X-ray findings were normal (Fig S5D).

A nasopharyngeal swab specimen collected on the fifth day of hospital admission tested positive for SARS-CoV-2. She died on the sixth day of hospitalization, nine days after birth. Cause of death was noted as perinatal asphyxia with hypoxic-ischemic encephalopathy stage I with early onset sepsis with disseminated intravascular coagulation.

Potential exposure: Source of exposure to SARS-CoV-2 is unknown. Family members or the immediate caregiver were not tested.

**Case Ne09:**

A 4-day old boy, born at term by vaginal delivery at a hospital, was admitted with complaints of not feeding well. On examination during admission, he was afebrile, irritable, capillary refill time was <3 s, pulse was 144 beats/min and weighed 2.84 kg. His respiratory rate was 54 breaths/min (vesicular breath sound, no added sound). Chest X-ray showed bilateral ground-glass opacity indicative of pneumonia due to SARS-CoV-2 (Fig S5, B).

He was provisionally diagnosed with early onset neonatal sepsis and pneumonia, and was treated with ceftazidime, amikacin, dopamine and sodium bicarbonate and supplemental oxygen. A nasopharyngeal swab specimen collected on the second day of hospital admission tested positive for SARS-CoV-2. He died on the same day with the final diagnosis of early onset neonatal sepsis, pneumonia and severe metabolic acidosis (See Table S1 for arterial blood gas analysis).

Potential exposure: Source of exposure to SARS-CoV-2 is unknown. Family members or the immediate caregiver were not tested.

**Case Ne10:**

A 4-day old girl, born by C-section, was admitted due to ruptured myelomeningocele. On examination, she was ill-looking, afebrile, pulse was 132 beats/min and weighed 2.6 kg. Respiratory rate was 40 breaths/min (vesicular breath sound, no added sound). She was treated with ceftriaxone and gentamicin. A nasopharyngeal swab specimen collected on the second day of hospital admission tested positive for SARS-CoV-2 and therefore she was referred to a COVID-19 designated hospital for surgery after 4 days.

During a telephone follow-up at the age of 40 days, the family reported that the child was admitted at another hospital for 7 days, and ruptured myelomeningocele was surgically repaired; shunt operation for the associated hydrocephalus will be performed after 6 months.

Potential exposure: Nasopharyngeal swab specimen collected from the mother the same day as that of the child tested positive for SARS-CoV-2. The mother was asymptomatic.

**Case Ne11:**

A 4-day old girl, born at term by vaginal delivery at home, was admitted with history of fever and respiratory distress. On examination, the baby was febrile (100^o^F), dyspnoeic, lethargic, had a pulse of 120 beats/min, and weighed 3.05 kg. Respiratory rate was 60 breaths/min (vesicular breath sound, no added sound). The child was diagnosed with early onset neonatal sepsis and was treated with ceftazidime, amikacin and supplemental oxygen.

A nasopharyngeal swab specimen collected on the second day of hospital admission tested positive for SARS-CoV-2. The child was referred to a COVID-19 designated hospital after 3 days and during a telephone follow-up at the age of 63 days, the child was at home and appeared to be healthy.

Potential exposure: Source of exposure to SARS-CoV-2 is unknown. Family members or the immediate caregiver were not tested.

**Case Ne12:**

A 4-day old boy was admitted with complaints of respiratory distress and passage of loose watery stool for 2 days. On general examination, he was ill-looking, afebrile, icteric, pulse was 100 beats/min and weighed 2.5 kg. Respiratory rate was 40 breaths/min (vesicular breath sound, no added sound). The child was diagnosed with early onset neonatal sepsis and neonatal jaundice and was treated with ceftazidime, and phototherapy.

A nasopharyngeal swab specimen collected on the second day of hospital admission tested positive for SARS-CoV-2. He was referred to a COVID-19 designated hospital after 3 days. The child was lost to follow up, and long-term outcome is unknown.

Potential exposure: Source of exposure to SARS-CoV-2 is unknown. Family members or the immediate caregiver were not tested.

**Case Ne13:**

A 4-day old boy, born at term by vaginal delivery at home was admitted with the complaints of delayed cry after birth and history of convulsion. During admission, he was lethargic, afebrile, icteric, poor reflexes, pulse was 120 beats/min and weighed 3.5 kg. Respiratory rate was 40 breaths/min (vesicular breath sound, bilateral crepitations). His provisional diagnosis was perinatal asphyxia with hypoxic-ischemic encephalopathy stage 2. After admission, the child developed fever, features of sepsis and respiratory rate increased to 68 breaths/min. Chest X-ray showed few patchy opacities in the right lower perihilar region (other infiltrates); the left lung field appeared normal (Fig S5, E), indicating non-specific inflammatory lesion in right lower zone. He was treated with ceftazidime, amikacin, meropenem, netilmicin, piperacillin+tazobactam, furosemide, dobutamine and phenobarbitone and supplemental oxygen.

A nasopharyngeal swab specimen collected on the nineth day of hospital admission tested positive for SARS-CoV-2. After 11 days of hospitalization, the child was referred to a COVID-19 designated hospital with perinatal asphyxia with HIE stage 2, EONS and pneumonia. The child was lost to follow up and final health outcome is unknown.

Potential exposure: Nasopharyngeal swab specimen collected from the mother the same day as that of the child tested positive for SARS-CoV-2. Details about the mother’s symptoms are unknown.

**Case Ne14:**

A 5-day old girl, born at term by C-section, was admitted with complaints of fever and not feeding well for 1 day, two episodes of convulsion, and yellowish coloration of skin and sclera. On general examination, the child was afebrile, moderately anaemic, icteric up to thigh, capillary refill time was <3 s, pulse was 128 beats/min, and weighed 3 kg. Respiratory rate was 40 breaths/min (vesicular breath sound, bilateral crepitations). Provisional diagnosis of the child was early onset neonatal sepsis and jaundice. She was treated with ceftazidime, amikacin and phenobarbitone, and supplemental oxygen.

A nasopharyngeal swab specimen collected on the fourth day of hospital admission tested positive for SARS-CoV-2. The child was referred to a COVID-19 designated hospital after 5 days. During a telephone follow-up at the age of 49 days, the child was reported to be at home and appeared healthy.

Potential exposure: Both parents were symptomatic for COVID-19 (reported cough and chest pain) but were not tested for SARS-CoV-2.

**Case Ne15:**

An 8-day old boy, born at term by C-section, was admitted with complaints of dribbling of urine and abdominal distension since birth. On examination, the child was alert, active, afebrile, moderately anaemic, icteric, had a pulse of 132 beats/min, and weighed 2.5 kg. Respiratory rate was 34 breaths/min (vesicular breath sound, no added sound). The child was provisionally diagnosed with acute kidney injury due to obstructive uropathy and was treated with ceftazidime, meropenem, sodium bicarbonate, calcium gluconate.

A nasopharyngeal swab specimen collected on the second day of hospital admission tested positive for SARS-CoV-2. The child was referred to a COVID-19 designated hospital after 4 days and during a telephone follow-up at the age of 63 days, the family reported that they were seeking care at another hospital and that the child was still sick.

Potential exposure: Source of exposure to SARS-CoV-2 is unknown. Family members or the immediate caregiver were not tested.

**Case Ne16:**

An 8-day old boy, born at term by C-section, was admitted with history of fever and not feeding well. On examination, he was moderately active and febrile (101^o^F); capillary refill time was <3 s, pulse was 130 beats/min and weighed 2.65 kg. Respiratory rate was 41 breaths/min (vesicular breath sound, no added sound). The child was provisionally diagnosed with late onset neonatal sepsis and was treated with ceftazidime and amikacin.

A nasopharyngeal swab specimen collected on the second day of the admission tested positive for SARS-CoV-2. The child was referred to a COVID-19 designated hospital after 2 days of hospital stay. During a telephone follow-up at the age of 70 days, the family reported that the child was at home and appeared healthy.

Potential exposure: Source of exposure to SARS-CoV-2 is unknown. Family members or the immediate caregiver were not tested.

**Case Ne17:**

A 14-day old boy, born by C-section, was admitted with history of dribbling of urine, crying during micturition, constipation and abdominal distension for 4 days and bilateral talipes equinovarus. On examination during admission, the child was febrile (100^o^F), ill-looking, lethargic, icteric, and weighed 2.5 kg. Respiratory rate was 25 breaths/min (vesicular breath sound, no additional sound). He was diagnosed with posterior urethral valve with bilateral hydroureteronephrosis (grade-3), urinary tract infection with bilateral talipes equinovarus and was treated with ceftazidime, amikacin, meropenem, metronidazole, furosemide, calcium gluconate and sodium bicarbonate.

A nasopharyngeal swab specimen collected on the sixth day of hospital admission tested positive for SARS-CoV-2. The child was referred to a COVID-19 designated hospital after 7 days. During the telephone follow-up, the family reported death of the child at the age of 28 days.

Potential exposure: Source of exposure to SARS-CoV-2 is unknown. Family members or the immediate caregiver were not tested.

**Case Ne18:**

A 19-day old boy, born by C-section, was admitted with respiratory distress and convulsion. He was admitted at another hospital for two days before admission at DSH. On general examination, the child was afebrile, dyspnoeic, capillary refill time was <3 s, pulse was 124 beats/min, weighed 2.6 kg. Respiratory rate was 40 breaths/min (vesicular breath sound, no added sound). With a provisional diagnosis of late onset neonatal sepsis, the child was treated with meropenem, amikacin, aminophylline, phenobarbitone and supplemental oxygen. Further examination revealed congenital heart disease with metabolic acidosis (see Table S1 for arterial blood gas analysis).

A nasopharyngeal swab specimen collected on the fourth day of hospital admission tested positive for SARS-CoV-2. The patient was referred to a COVID-19 designated hospital after 5 days. During telephone follow-up, the family reported that they did not seek care at any other hospital and the child died at the age of 25 days.

Potential exposure: Source of exposure to SARS-CoV-2 is unknown. Family members or the immediate caregiver were not tested.

**Case Ne19:**

A 22-day old boy, born at term by C-section, was admitted with complaints of fever for 3 days, respiratory distress and not feeding well for 1 day. During admission, he was lethargic, dyspnoeic, had a pulse of 140 beats/min, and weighed 3.1 kg. His respiratory rate was 42 breaths/min (vesicular breath sound, no added sound).

A nasopharyngeal swab specimen collected on the second day of hospital admission tested positive for SARS-CoV-2. He was treated with ceftazidime, amikacin and supplemental oxygen and was referred to a COVID-19 designated hospital after 3 days with the diagnosis of late onset neonatal sepsis. During a telephone follow-up at the age of 49 days, the family reported seeking care at two other hospitals after DSH, and that the child was currently at home and appeared healthy.

Potential exposure: Nasopharyngeal swab specimen collected from the mother the same day as that of the child tested positive for SARS-CoV-2. The mother was asymptomatic.

**Case Ne20:**

A 22-day old boy was admitted with history of fever and convulsion for 2 days. He was hospitalized in another hospital prior to admission at DSH. On examination, his pulse was 130 beats/min, temperate was 98.6 F, and weighed 3.9 kg. Convulsion was focal and persisted for <5 min. Respiratory rate was 30 breaths/min, (vesicular breath sound, no added sound). He was diagnosed with late-onset sepsis and was treated with ceftriaxone, amikacin and phenobarbitone.

A nasopharyngeal swab specimen collected after 6 days of hospitalization tested positive for SARS-CoV-2. The child was discharged after 8 days of hospitalization, and during a telephone follow-up at the age of 81 days, the family reported that the child was at home and appeared healthy.

Potential exposure: Source of exposure to SARS-CoV-2 is unknown. Family members or the immediate caregiver were not tested.

**Case Ne21:**

A 23-day old boy, born at term by normal vaginal delivery at a hospital, was admitted with history of convulsion for 6 days and not feeding well for 2 days. On examination during admission, the child was afebrile, ill-looking, had generalized tonic-clonic seizure. His heart rate was 160 beats/min, weight was 3.2 kg, and respiratory rate was 56 breaths/min (vesicular breath sound, no added sound). On cardiovascular examination, the child had a pansystolic murmur and was diagnosed with congenital heart disease with late onset neonatal sepsis.

He was treated with ceftazidime, meropenem and furosemide, calcium gluconate, dobutamine and supplemental oxygen. A nasopharyngeal swab specimen collected on the third day of hospital admission tested positive for SARS-CoV-2. After 4 days, the child was shifted to a COVID-19 designated hospital, where he died after 3 days, at the age of 30 days.

Potential exposure: Source of exposure to SARS-CoV-2 is unknown. Family members or the immediate caregiver were not tested.

**Case Ne22:**

A 23-day old boy, born at term by vaginal delivery at home, was admitted with history of fall from height, epistaxis and bleeding with frothy secretion from mouth. During admission, he was alert, afebrile, pulse was 120 beats/min, and weighed 4 kg. No active bleeding, no source of bleeding was noted during admission. Respiratory rate was 38 breaths/min (vesicular breath sound, no added sound). He was treated with mupirocin 2% topical ointment.

A nasopharyngeal swab specimen collected on the second day of hospital admission tested positive for SARS-CoV-2. He was referred to a COVID-19 designated hospital after 4 days. During a follow-up at 57 days, the family reported that they did not seek care at any other hospital, and that the child was at home and overall doing well.

Potential exposure: The mother was symptomatic for COVID-19. Nasopharyngeal swab specimen of the mother, collected on the same day as the child, also tested positive for SARS-CoV-2.

**Case Ne23:**

A 23-day old boy was admitted with the complaints of fever and cough for 2 days and respiratory distress for 1 day. During admission, he was febrile (100.2^o^F), pulse was 120 beats/min, and weighed 3.4 kg. His respiratory rate was 40 breaths/min (vesicular breath sound, bilateral crepitations). His diagnosis was pneumonia and was treated with ceftazidime, amikacin and supplemental oxygen. Chest X-ray findings were normal (Fig S5C).

A nasopharyngeal swab specimen collected on the second day of hospital admission tested positive for SARS-CoV-2. He was referred to a COVID-19 designated hospital after 4 days at DSH. The child was lost to follow up and final health outcome is unknown.

Potential exposure: Nasopharyngeal swab specimen of the mother, collected on the same day as the child, also tested positive for SARS-CoV-2.Details about the mother’s symptoms are unknown.

**Case Ne24:**

A 25-day old girl, born at term by C-section, was admitted with the complaints of respiratory distress for 2 days and not feeding well for 1 day. On general examination during admission, the child was febrile (100^o^F), dyspnoeic, heart rate was 160 beats/min, and weighed 3.2 kg. Respiratory rate was 66 breaths/min (vesicular breath sound, bilateral crepitations). Provisional diagnosis was pneumonia with late onset neonatal sepsis and the child was treated with ceftriaxone, amikacin and supplemental oxygen.

A nasopharyngeal swab specimen collected on the first day of hospital admission tested positive for SARS-CoV-2. The child was referred to a COVID-19 designated hospital after two days at DSH. During a telephone follow-up at the age of 82 days, the family reported that the child was at home and appeared healthy.

Potential exposure: The mother reported history of cough during the weeks prior to hospitalization of the child, but was not tested for SARS-CoV-2.

**Case Ne25:**

A 28-day old girl, born at term by C-section, was admitted with history of cough and fever for 2 days. On general examination during admission, she was ill looking, lethargic, afebrile, capillary refill time was <3 s, pulse was 130 beats/min and weighed 3.2 kg. Respiratory rate was 44 breaths/min (vesicular breath sound, bilateral crepitations). The provisional diagnosis was late onset neonatal sepsis with pneumonia, and she was treated with meropenem and amikacin.

A nasopharyngeal swab specimen collected on the third day of hospital admission tested positive for SARS-CoV-2. This child’s family left after 4 days to seek care at another hospital, where she was admitted for 6 days. During a telephone follow-up at the age of 77 days, the child was at home and was reported to be healthy.

Potential exposure: Nasopharyngeal swab specimen of the mother, collected on the same day as the child, also tested positive for SARS-CoV-2.The mother was asymptomatic.

**Case Ne26:**

The hospital file of this child was lost, and clinical details of symptoms and treatment could not be recovered. The following history was collected by interviewing the mother and from laboratory records.

A 28-day boy, born at term by C-section, was admitted with history of fever and cough. A nasopharyngeal swab specimen collected on the third day of admission tested positive for SARS-CoV-2. The child was discharged after 6 days of hospitalization and during a telephone follow-up at the age of 102 days, the family reported that the child was at home and healthy.

Potential exposure: Source of exposure to SARS-CoV-2 is unknown. Family members or the immediate caregiver were not tested.
