# Supplementary material for: The Direct and Indirect Impact of SARS-CoV-2 Infections on Neonates: A Series of 26 Cases in Bangladesh
Source: Pediatr Infect Dis J. 2020 Oct 6;39(12):e398–405. doi: 10.1097/INF.0000000000002921 (PMC7654949; doi:10.1097/INF.0000000000002921)
Supplement: Supplementary file 2 [file inf-39-e398-s002.docx]

# **Supplemental Digital Content 2.** Summary of hematologic, biochemical and molecular laboratory results

Blank cells indicate tests not conducted.

Abbreviations: CT: cycle threshold; Blood C/S: blood culture and sensitivity; S: Serum; CBC: Complete blood count; RBC: Red blood cell count; Hb: haemoglobin; WBC: white blood cell count; CRP: C-reactive protein; BE (B): base excess (blood); BE (ecf): base excess (extracellular fluid); GISAID: <https://www.gisaid.org/>
