# Supplementary material for: The Direct and Indirect Impact of SARS-CoV-2 Infections on Neonates: A Series of 26 Cases in Bangladesh
Source: Pediatr Infect Dis J. 2020 Oct 6;39(12):e398–405. doi: 10.1097/INF.0000000000002921 (PMC7654949; doi:10.1097/INF.0000000000002921)

# **Supplemental Digital Content 3.** Phylogenetic tree of nine SARS-CoV-2 genomes from neonates and other sequenced Bangladeshi genomes

The figure was rendered using Nextstrain Asia build on 21 July 2020 and includes 3679 SARS-CoV-2 sequences from Asia and 223 sequences from Bangladesh (shown in light blue circles). All nine sequences from neonatal cases are shown in red circles. The neonatal cases cluster with other Bangladesh SARS-CoV-2 sequences, representing instances of community transmission. The strains belonged to clades 20A, 20B and 20C.


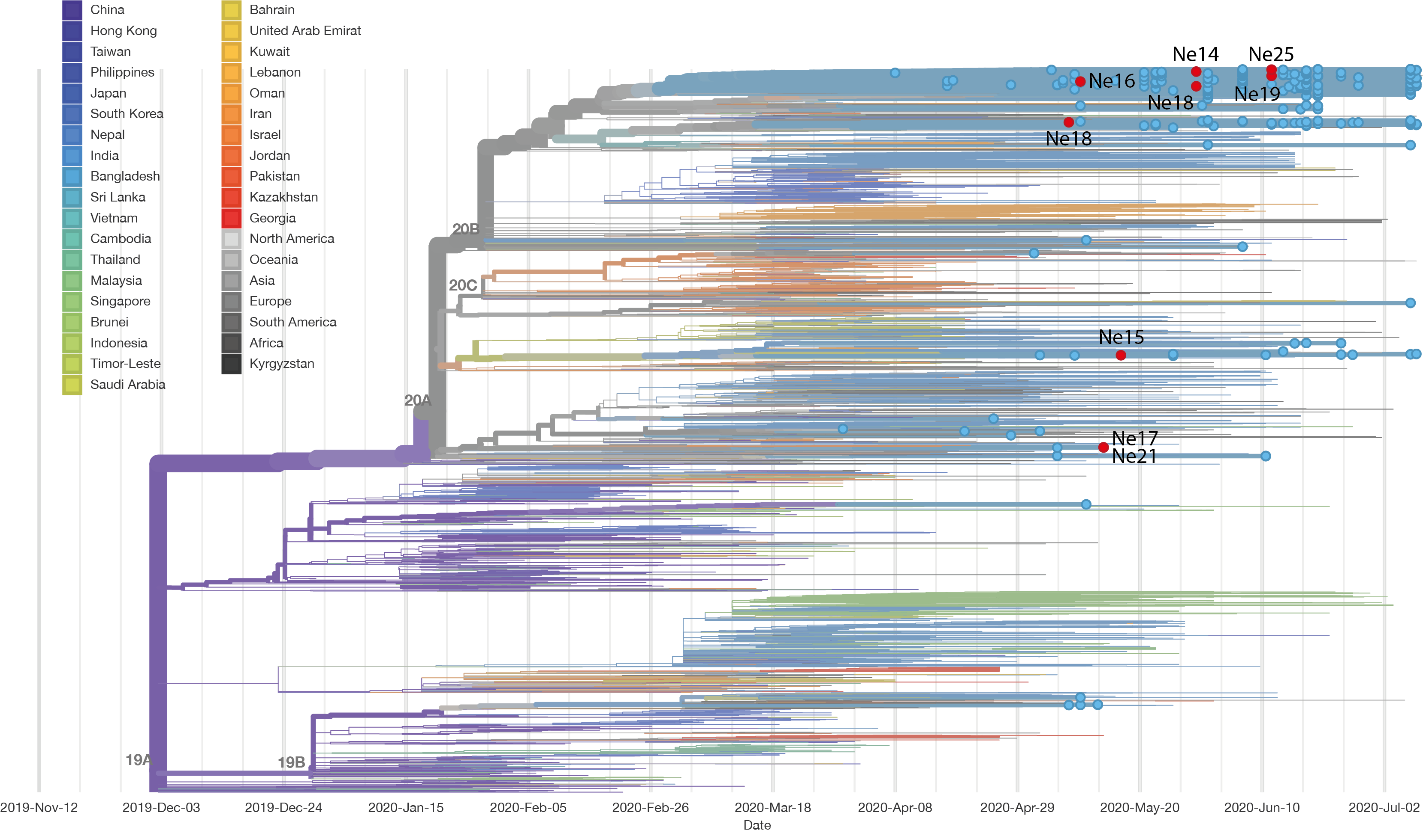

Supplement: Supplementary file 3 [file inf-39-e398-s003.docx]
