# Supplementary material for: The Direct and Indirect Impact of SARS-CoV-2 Infections on Neonates: A Series of 26 Cases in Bangladesh
Source: Pediatr Infect Dis J. 2020 Oct 6;39(12):e398–405. doi: 10.1097/INF.0000000000002921 (PMC7654949; doi:10.1097/INF.0000000000002921)
Supplement: Supplementary file 4 [file inf-39-e398-s004.docx]

# **Supplemental Digital Content 4.** Geographical distribution of the neonates with SARS-CoV-2 infections

**
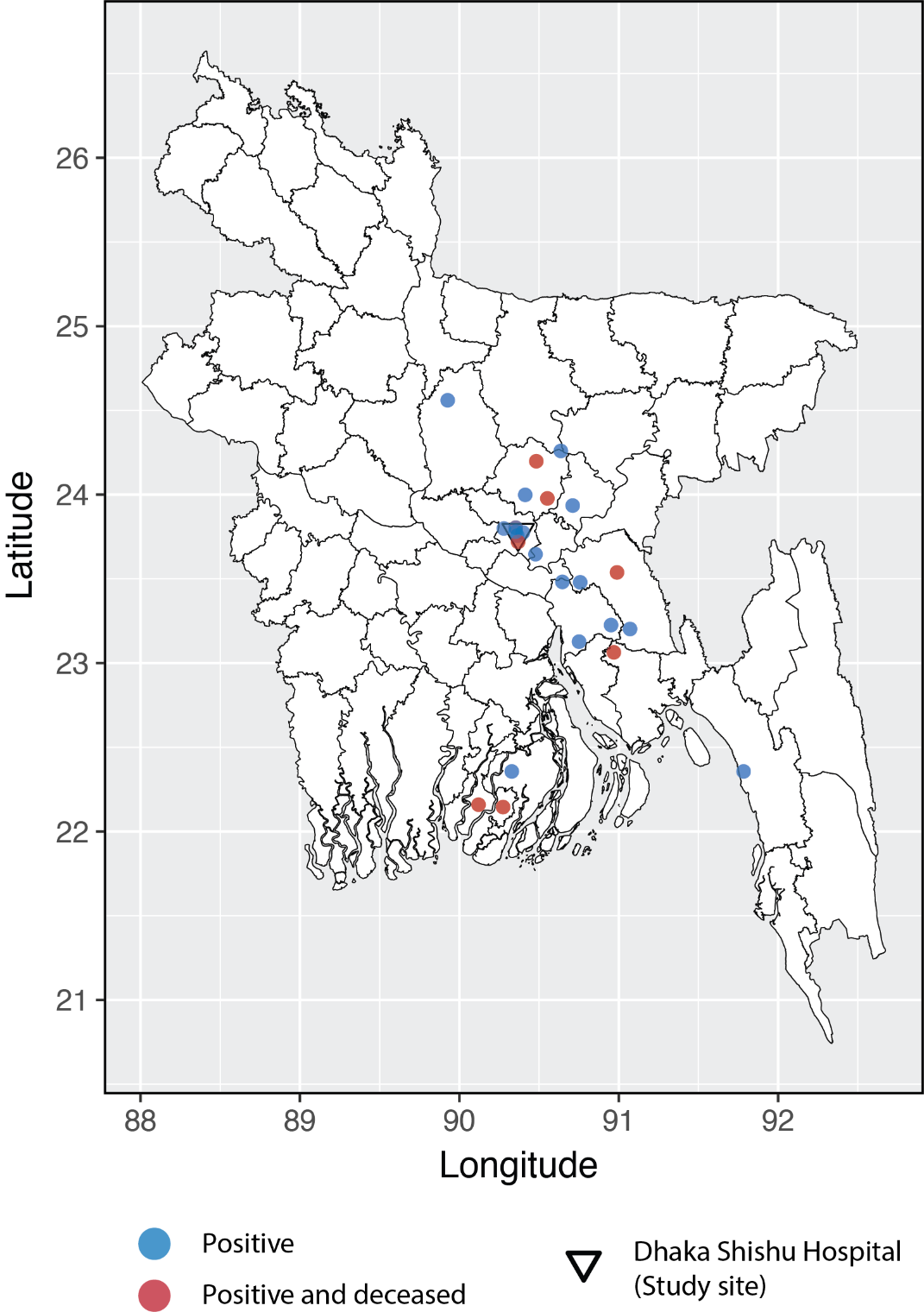
**
